# Obstetric fistula in Niger: 6-month postoperative follow-up of 384 patients from the Danja Fistula Center

**DOI:** 10.1007/s00192-017-3375-7

**Published:** 2017-06-09

**Authors:** Itengre Ouedraogo, Christopher Payne, Rahel Nardos, Avril J. Adelman, L. Lewis Wall

**Affiliations:** 1 The Danja Fistula Center, Danja, Niger; 2 The Worldwide Fistula Fund, Chicago, IL USA; 3 Department of Obstetrics and Gynecology, Oregon Health and Sciences University, Portland, OR USA; 4 Division of Biostatistics, Washington University School of Medicine, St. Louis, MO USA; 5 Department of Anthropology, Washington University in St. Louis, Campus Box 1114, One Brookings Drive, St. Louis, MO 63110 USA; 6 Department of Obstetrics and Gynecology, Washington University School of Medicine, St. Louis, MO USA

**Keywords:** Obstetric fistula, Vesicovaginal fistula, Surgery, Niger, Obstructed labor

## Abstract

**Introduction and hypothesis:**

The impoverished West African country of Niger has high rates of obstetric fistula. We report a 6-month postoperative follow-up of 384 patients from the Danja Fistula Center and assess factors associated with operative success or failure.

**Methods:**

The medical records of 384 women who had completed a 6-month follow-up after fistula surgery were reviewed. Cases were categorized as “easy,” “of intermediate complexity,” or “difficult” based on a preoperative points system. Data were analyzed using simple chi-squared statistics and logistic regression.

**Results:**

The patients were predominantly of Hausa ethnicity (73%), married young (average 15.9 years), had teenage first pregnancies (average first delivery 16.9 years), and experienced prolonged labor (average 2.3 days) with poor outcomes (89% stillbirth rate). The average parity was four. Patients commonly developed their fistula during their first delivery (43.5%), but over half sustained a fistula during a subsequent delivery (56.5%). Prior fistula surgery elsewhere (average 1.75 operations) was common. The overall surgical success (“closed and dry”) was 54%. When the 134 primary operations were analyzed separately, the overall success rate was 80%. Increasing success was seen with decreasing surgical difficulty: 92% success for “easy” cases, 68% for “intermediate” cases, and 57% success for “difficult” cases. Success decreased with increasing numbers of previous attempts at surgical repair.

**Conclusions:**

These data provide further evidence that clinical outcomes are better when primary fistula repair is performed by expert surgeons in specialist centers with the support of trained fistula nurses.

## Introduction

An obstetric fistula is an abnormal passageway between the vagina and the urinary and/or gastrointestinal tract arising from obstetric trauma [1]. In sub-Saharan Africa, these injuries most commonly arise from a crush injury to the vesicovaginal septum during prolonged obstructed labor [1]. These injuries are completely preventable provided the diagnosis of obstructed labor is made early and prompt intervention takes place before extensive ischemia has occurred. However, poorly developed systems of maternal healthcare in West Africa prevent many women from accessing emergency obstetric services in a timely fashion after labor becomes obstructed. This often leads to catastrophic injuries [2, 3]. Obstetric fistula is largely a disease of poverty, and West Africa is extremely poor [4].

Niger is a landlocked West African country located directly north of Nigeria. It is the world’s poorest nation, listed at 187 out of 187 ranked countries in the 2014 United Nations Human Development Index [5]. According to the UN’s Multidimensional Poverty Index (MPI) which identifies multiple deprivations in education, health and living standards in the same household, 90% of the population of Niger is “multidimensionally poor” [5]. Maternal mortality is high, with a maternal mortality ratio of 630 maternal deaths per 100,000 live births [6]. The overall rate of cesarean delivery is only 1%, far below the absolute minimum required to deal with obstructed labor and other maternal health needs [7]. The combination of dire poverty, extremely high rates of population growth, and poor access to reproductive healthcare means that obstetric fistulas are common in Niger.

Danja is a small village located 15 km south of Maradi, the third largest city in Niger, in the middle of Nigerian Hausaland. The Hausa are one of the largest ethnic groups in West Africa, whose traditional emirates were bifurcated between the British and the French during the colonial partition of West Africa [8]. Obstetric fistulas are prevalent on both sides of the Niger/Nigeria border [9–18]. A newly constructed hospital with 42 beds, an outpatient clinic, a patient hostel, administrative offices, and a dedicated fistula operating theater was opened in early 2012 in the grounds of the *Centre de Sante et de Leprologie* (CSL), a leprosy hospital established by Serving in Mission (SIM), a Christian missionary organization that has been working in Niger since 1924 [19]. The Danja Fistula Center is the only fistula center in Niger with an expert fistula surgeon trained to International Federation of Gynecology and Obstetrics (FIGO) criteria in residence. Patient services are provided free of charge. Over 90% of the funds for the operation of the center come from The Worldwide Fistula Fund, a not-for-profit public charity based in Chicago, IL, USA.

Numerous case series of obstetric fistulas have been published in the literature, but they invariably present only short-term results, usually at the time of hospital discharge or within a few weeks thereafter [11–23]; papers presenting data with six-month follow-up are rare [24]. This study was undertaken to review our results 6 months after fistula repair in a rural setting in West Africa.

## Materials and methods

A retrospective, observational, cohort study was carried out utilizing the case records of 384 women seen for care at the Danja Fistula Center between January 2013 and July 2014. These case records were examined to determine the outcomes of surgery. This study was approved by the medical director of the Danja Fistula Center as involving minimal risk, as it was a confidential retrospective review of medical records not requiring further interventions. Fistula cases had been categorized as “easy,” of “intermediate” complexity, or “difficult” at the time of examination in the operating room prior to the commencement of surgery, and the complexity score was recorded in the patient’s chart. Complexity was assessed using a points system (Table 1) modified from a system originally proposed by Arrowsmith [25]. This system incorporates factors commonly believed to be associated with surgical outcomes in fistula repair: degree of involvement of the continence mechanism of the urethra and bladder neck in the fistula, degree of scarring present, and the number of previous attempts at surgical repair [26, 27]. The type and distribution of the obstetric fistulas encountered are given in Table 2. Patients had an average of 1.75 prior fistula operations, with a median of 1.0 and a range from 0 to 10 prior attempted repairs. The vast majority of the previous repairs (73%) had been carried out at other hospitals in Nigeria or Niger.

**Table 1:** Points system for scoring difficulty of surgical repair

Factor	Points awarded			
	0	1	2	3
Involvement of urethra or bladder neck	None	Mild	Significant involvement	Total loss
Degree of scarring	None	Mild	Moderate	Severe
Number of previous surgeries	0	1	2	3 or more

Surgical difficulty: “easy” 0–2 points, “Intermediate” 3–5 points, “difficult” 6–9 points

**Table 2:** Type and distribution of obstetric fistulas

Type of fistula	Number	Percentage of total
Urethrovaginal fistula	61	17
Juxtaurethral fistula	118	33
Circumferential fistula	40	11
Midvaginal fistula	40	11
Juxtacervical fistula	69	19
Vesicocervical fistula	14	4
Large fistula^a^	15	4
Ureterovaginal fistula	5	1
Missing data/other	22	6
Total	362	100

^a^Fistula with very extensive loss of bladder and urethra comprising at least two thirds of the anterior vaginal wall

Fistula surgery was performed under spinal anesthesia using a transvaginal approach in 98% of patients. Postoperatively the bladder was emptied by free drainage using an indwelling transurethral catheter for 7 to 10 days (average 9 days, range 2 to 29 days). Prophylactic intravenous gentamicin at 5 mg/kg body weight was given before attempted vesicovaginal fistula repair. Patients undergoing surgery for a rectovaginal fistula (either alone or in combination with repair of a vesicovaginal fistula) were given 1 g of ceftriaxone and 500 mg of metronidazole intravenously as prophylaxis. The bladder layer was closed using 3-0 polyglactin 910 sutures (Vicryl; Ethicon, Somerville, NJ) and the vaginal layer was closed with 2-0 polyglactin 910 (Vicryl). Urethral reconstruction and ureteral reimplantation was done using 5-0 polyglactin 910 sutures (Vicryl, Ethicon, Somerville, NJ).

Patients with persistent fistula at the time of discharge were classified as “failed” and redo surgery was scheduled when appropriate. A delayed follow-up evaluation was performed in all other patients in this study. Patients who were enrolled in our 3-month reintegration program were assessed after completion. Others were asked to return at 6 months. Patients were reimbursed for the cost of their transportation as an incentive to return. Patients were advised not to become pregnant without having a follow-up examination and counseling. All of the patients eligible for inclusion in this study returned 6 months after surgery; none was lost to follow-up.

The outcomes of surgery were assessed using a blue dye test and an interview performed by the surgeon and lead author—generally in the patient’s native language but with the aid of a translator when necessary. The interview was unstructured and the goal was to determine if any incontinence was present and to assess the likely cause. A pelvic examination was then performed. After inspection a Foley catheter was placed, the residual urine volume was measured, and the bladder was filled with blue dye to a comfortable volume. The patients were examined for the presence of persistent or recurrent fistulas. If no fistula was present, a cough test was performed to detect stress incontinence. Leakage occurring with urgency was also noted during bladder filling.

The analysis team first created two variables from the collected data: “closed” versus “open” (failed repair) with respect to fistula status, and “dry” versus “wet” with respect to continence status. This allowed separation of surgical failure to close the fistula from successful fistula closure with persistent incontinence from a cause unrelated to fistula [28]. The ultimate classification decision was made by the surgeon based on his best judgment after the examination and interview. The following variables were kurtotic (non-normally sharp peak to their distribution) and log transformation was used to reduce the kurtosis: age at first delivery, age at first marriage, duration of labor, and time with catheter. All analyses were performed on each variable, first by closed versus open outcome, and then by dry versus wet outcome. The chi-squared test was used to evaluate the relationship between surgical success and method of delivery (spontaneous vaginal delivery, instrumental vaginal delivery, or cesarean delivery). Logistic regression analysis was used to evaluate the relationships between surgical success and difficulty of repair, number of previous repairs, duration of the fistula in years, duration of labor, number of deliveries, and time with catheter after surgery.

## Results

A total of 384 patients were included in this study. The patients were overwhelmingly of Hausa ethnicity (280 women, 73%), with significant numbers of Fulani women (42, 11%) and Tuareg women (41, 11%). This mirrors the ethnic mix of this part of Niger. There were also a small number of Kanuri women (17, 4%) from the far east of Niger/Nigeria, three Zarma women (1%) from the far west of Niger, and one Arab woman who had crossed the Sahara from North Africa via Agedez. The average patient age was 29.3 years (median 29.0 years, mode 30 years, range 14 to 65 years). The average age at first marriage was 15.9 years (median 15 years, mode 15 years, range 12 to 28 years) and the average age at first delivery was 16.9 years (median 16.0 years, mode 16.0 years, range 14 to 28 years). As a group, these women were poorly educated: 94% were illiterate and only 23 had any formal education. One patient had been to university. Half of the patients (185 women) were being supported financially by their husband and 44% (164 women) were being supported financially by their parents; 4% (15 women) were dependent on their own support and 2% (6 women) had other means of support. With respect to marital status, 204 women (55%) were married, 94 women (25%) described themselves as separated, 61 women (17%) were divorced, and 11 women (3%) were widowed (data were missing on 14 women).

On average, patients had suffered from an obstetric fistula for 6.4 years (median 4 years, range less than 1 year to 40 years). The average duration of labor for the index delivery that resulted in a fistula (recorded in 363 women) was 2.3 days (median 2 days, mode 2 days, range 1 to 7 days). Pregnancy outcomes for the index delivery were bleak, as shown in Table 3. Of 364 women for whom data were recorded, only 64 (18%) delivered at home. The rest (300 women, 82%) delivered at a health center, but too late. Of these 364 women, 175 (48%) had a spontaneous vaginal delivery, 59 (16%) underwent operative vaginal delivery, and 130 (36%) underwent cesarean section.

**Table 3:** Outcome of index pregnancy in which fistula occurred

Outcome	Number	Percentage of total
Stillbirth	323	88.7
Live-born	33	9.1
Neonatal death in first week	8	2.2
Missing data/other	20	5.0
Total	384	100.0

Although the most common presentation was the development of an obstetric fistula during a woman’s first delivery, high parity was common in this series of patients. The average parity at the time of fistula occurrence among this group of patients was 4, with a median parity of 3, and a range of parity from 1 to 14. Table 4 shows the distribution of the index deliveries at which the fistula occurred. Overall reproductive outcome was dismal. These women with obstetric fistulas had an average of 1.6 living children, with a median of one living child (mode no living children, range 0–10 living children); however, 179 women had no living children at all.

**Table 4:** Index delivery at which fistula occurred

Index delivery	Number of women	Percentage of total
1	167	43.5
2	34	8.9
3	33	8.6
4	19	4.9
5	21	5.5
6	10	2.6
7	17	4.4
8	26	6.8
9	11	2.9
10	8	2.1
11	10	2.6
12	4	1.0
13	2	0.5
14	2	0.5
Missing data	20	5.2
Total	384	100.0

The mix of surgical cases was skewed. Only 25% of the cases (98 women) were classified as easy, 26% (99 women) were classified as intermediate difficulty, and 49% (187 women) were classified as difficult. Only ten operations were carried out under general anesthesia (three of these following failed attempts to induce spinal anesthesia). Only eight operations were carried out using a transabdominal route. Overall surgical outcomes are given in Table 5. In 54% of patients surgery was successful, with an outcome of “closed and dry,” meaning that the fistula was closed and the patient was continent, in 30% of patients closure failed, meaning that the fistula had not sealed following surgery and thus remained open, and in 16% of patients the outcome was “closed but wet,” meaning that the fistula had been closed and there was no extraurethral urine loss but some transurethral urine loss persisted [26]. There is a general consensus among fistula surgeons that the first surgical repair is the most likely to be successful [18–23]. Therefore outcomes in the 134 patients who underwent primary surgical repair at the Danja Fistula Center are shown separately in Table 6, categorized by the difficulty of surgery assessed using the preoperative points system presented in Table 1. The success rate (closed and dry) in these patients was excellent, at 80%.

**Table 5:** Overall outcomes of fistula surgery within each category of difficulty

Difficulty of repair	Number of cases	Closed and dry	Closed but wet	Failed repair
Easy	98	88 (90.0%)	3 (3.1%)	7 (7.1%)
Intermediate	99	54 (54.5%)	22 (22.2%)	23 (23.2%)
Difficult	187	65 (34.8%)	38 (20.3%)	84 (44.9%)
Total	384 (100%)	207 (53.9%)	63 (16.4%)	114 (29.7%)

**Table 6:** Outcome of primary surgery by degree of difficulty in 134 patients

Degree of difficulty	Outcome			Total for each degree of difficulty
	Closed and dry	Closed but wet	Failed closure	
Easy	71 (92%)	2 (3%)	4 (5%)	77 (58%)
Intermediate	23 (68%)	4 (12%)	7 (21%)	34 (25%)
Difficult	13 (57%)	1 (4%)	9 (39%)	23 (17%)
Total for each outcome	107 (80%)	7 (5%)	20 (15%)	134 (100%)

The likelihood of a closed and dry outcome decreased substantially with increasing difficulty of repair (logistic Wald odds ratio estimate 0.371, 95% CI 0.214–0.642, *p* < 0.0001, for a closed outcome; and 0.444, 95% CI 0.270–0.730, *p* < 0.0001, for a dry outcome). The likelihood of a closed and dry outcome also decreased with an increasing number of previous repair attempts (logistic Wald odds ratio estimate 0.754, 95% CI 0.673–0.844, *p* < 0.0001, for a closed outcome; and 0.617, 95% CI 0.537–0.710, *p* < 0.0001, for a dry outcome), and with increasing log of time with catheter after surgery (logistic Wald odds ratio estimate 0.3418, 95% CI 0.227–0.770, *p* < 0.0051, for a closed outcome; and 0.457, 95% CI 0.266–0.786, *p* < 0.0046, for a dry outcome). The likelihood of only a dry outcome decreased with an increasing number of deliveries (logistic Wald odds ratio estimate 1.118, 95% CI 1.048–1.193, *p* < 0.0007). The association between a closed outcome and the number of deliveries was only marginally significant (logistic Wald odds ratio estimate 1.072, 95% CI 0.999–1.150, *p* < 0.0536).

The method of delivery in the index pregnancy (simple vaginal delivery, cesarean section, or instrumental vaginal delivery) was also significantly associated with fistula status (closed versus open; chi-squared = 9.18, *p* = 0.0102) and with the ultimate outcome (dry versus wet; chi-squared = 8.14, *p* = 0.0171). The differences can be explained by more favorable outcomes in women who had a simple vaginal delivery as opposed to those who required an instrumental vaginal delivery or cesarean section. The following associations were marginally significant or not significant: fistula status (closed versus open) and ultimate outcome (dry versus wet) in relation to duration of fistula in years (logistic Wald odds ratio estimate 1.000, 95% CI 0.971–1.030, *p* < 0.9903; and 0.974, 95% CI 0.947–1.001, *p* < 0.0545, respectively), and fistula status and ultimate outcome in relation to log of duration of labor in hours (logistic Wald odds ratio estimate 1.070, 95% CI 0.671–1.705, *p* < 0.7772; and 0.790, 95% CI 0.514–1.212, *p* < 0.2801, respectively).

## Discussion

The demographic characteristics of the patients seen at the Danja Fistula Center do not differ significantly from Nigerien fistula patients seen at other centers [11–18]. The Danja fistula patients are predominantly poor, illiterate women from rural areas, who typically marry early and commence their reproductive lives within a year of marriage. They receive inadequate obstetric care during their pregnancies, endure prolonged labors which generally last at least 2 days and typically end with the delivery of a stillborn fetus. Although most of these women ultimately deliver in a healthcare facility, the care they receive is substandard and generally “too little, too late.” Although half of these women develop a fistula as the result of obstructed labor in their first pregnancy, the others develop a fistula in a second, third, or later gestation. Indeed, two Danja patients developed their fistulas as a result of obstructed labor in their 14th pregnancy.

The Danja data also confirm the enormous suffering experienced by women with a fistula. The average Danja fistula patient had lived with her condition for nearly 6.5 years. These women have spent much of their time away from home and family searching for a cure, often undergoing surgery without success (65% of the Danja patients had had at least one prior attempt at repair); this phenomenon appears to be increasingly common among fistula patients in West Africa [17, 18]. The combination of the stigma and embarrassment caused by constant urinary loss as well as the strain on family ties caused by the incessant quest for therapy is shown in the high number of women who are divorced or separated (42%), or dependent upon their parents for social and economic support (44%). The quest for therapy—and the consequences of unsuccessful therapy in particular—becomes an increasing burden for these women which erodes both their financial and social resources [23, 24].

Most reports of the outcome of fistula surgery describe the condition of patients at the time of hospital discharge. It is clear that some patients with successful fistula closure at discharge experience failure of their surgical repair at some point after leaving hospital; our follow-up of patients at 6 months is therefore likely to be more representative of long-term outcomes. Browning and Menber reported a 6-month follow-up of 240 fistula patients that included 8 patients (2.1%) with a failed repair and 95 patients (24.3%) with persistent incontinence, but their series had a follow-up rate of only 62% and no data were given on the number of patients with a previously attempted fistula repair [24]. The overall surgical success rate of 54% in our series—defined as the fistula closed and the patient continent---is good, considering the high degree of difficulty and the large number of patients with previously attempted surgical repair. The degree of difficulty appears to be associated with increasingly severe damage to vaginal and bladder tissues. This interpretation of the data is supported by the significance of the relationship between the method of delivery and both successful surgical closure of the fistula and the ultimate outcome of wet versus dry. Patients who had had a simple vaginal delivery in the index pregnancy resulting in the fistula were more likely to have a favorable surgical outcome than women who had either an instrumental vaginal delivery or a cesarean section. We interpret this as representing greater tissue damage in deliveries requiring either operative vaginal delivery or cesarean section.

Analyzing the outcome of primary surgery in terms of the degree of difficulty (Table 6), the importance of expert care at the initial surgery is striking. After surgery, 80% of patients were closed and dry, including 92% of easy cases and 68% of intermediate cases, but only 57% of difficult cases. None of the fistula classification systems currently in use has good predictive accuracy for fistula closure, much less for continence after fistula repair [28]. The prognostic score used in this series to categorize cases as easy, of intermediate complexity, or difficult, appears to correlate well with surgical outcomes. Other fistula centers should consider prospective assessment and validation of this approach to see if these encouraging preliminary results hold up under further examination. Many authors have remarked that the first surgical repair is the most likely to be successful [20–23]. Our results provide further evidence that clinical outcomes are better when obstetric fistula surgery is performed in specialist centers by expert surgeons supported by trained fistula nurses [15, 24, 29, 30].

The strengths of this study include the large number of patients and the excellent follow-up, as no eligible patients were lost to follow-up. This can be attributed to the high level of personal, compassionate care provided at the center, the availability of food and shelter at a dedicated patient hostel, payment of transportation expenses for patients, and the intense desire of these fistula patients for future fertility. Many patients returned seeking advice regarding future fertility. This was particularly true of patients who were found to be “closed and dry.” Most of our patients were desirous of another pregnancy. The weaknesses of the study include failure to record each individual parameter of the points system separately in the hospital notes, failure to incorporate other fistula classification systems into the data collection process, lack of a fully standardized postoperative evaluation protocol, incomplete understanding of the type of incontinence in those patients who were “closed but wet,” and the lack of a separate patient-reported outcome measure. Reaching an accurate diagnosis in patients who were “closed but wet” requires more sophisticated urodynamic evaluation than is commonly available at African fistula centers. This is a subject for further investigation.

Our results suggest that efforts should continue to create high-volume centers of clinical excellence for patients with an obstetric fistula where this condition is epidemic. Such centers should collaborate better to understand, evaluate, and treat the problem of post-fistula incontinence. The ultimate goal should be to make such centers obsolete as the quality of obstetric services improves and the incidence of prolonged obstructed labor falls. During this process of healthcare system development, efforts should be redoubled to insure that patients with an obstetric fistula are treated by expert surgeons at the time of primary surgical repair.
